# An evaluation of overall survival in patients with newly diagnosed acute myeloid leukemia and the relationship with glasdegib treatment and exposure

**DOI:** 10.1007/s00280-020-04132-x

**Published:** 2020-09-03

**Authors:** Swan Lin, Naveed Shaik, Geoffrey Chan, Jorge E. Cortes, Ana Ruiz-Garcia

**Affiliations:** 1 Clinical Pharmacology, Global Product Development, Pfizer Inc, 10555 Science Center Dr, San Diego, CA 92121 USA; 2 Pfizer Oncology, Global Product Development, Pfizer Inc, Collegeville, PA USA; 3 Georgia Cancer Center, Augusta, GA USA; 4 Present Address: Metrum Research Group, San Diego, CA USA

**Keywords:** Acute myeloid leukemia, Exposure–response, Hedgehog, Overall survival, Smoothened inhibitor

## Abstract

**Purpose:**

Glasdegib, an oral inhibitor of the Hedgehog signaling pathway, is approved in the United States in combination with low-dose cytarabine (LDAC) to treat patients with newly diagnosed acute myeloid leukemia (AML) ineligible to receive intensive chemotherapy. This population pharmacokinetic/pharmacodynamic analysis characterized the time course of survival with glasdegib + LDAC relative to LDAC alone, and explored whether the differences in glasdegib exposure at the clinical dose of 100 mg once daily (QD) significantly affected overall survival (OS).

**Methods:**

Data from the BRIGHT AML 1003 trial in patients with AML were included in treatment–response (glasdegib + LDAC, *n* = 78; LDAC alone, *n* = 38) and exposure–response (glasdegib + LDAC, *n* = 75) analyses.

**Results:**

The analyses demonstrate that patients treated with glasdegib + LDAC (vs LDAC alone) at any time point during the study period were 58% less likely to die, translating to prolonging of median OS by ~ 5 months (hazard ratio 0.42 [95% confidence interval 0.28–0.66]). Variability in glasdegib exposures did not impact the risk of death. Additionally, potential covariates such as patient demographics, prior treatment with a hypomethylating agent, baseline safety laboratory values, and disease characteristics, did not impact the probability of OS.

**Conclusion:**

Together these results confirm that glasdegib + LDAC treatment (vs. LDAC alone) is associated with a significant survival benefit in patients with newly diagnosed AML, and that variability in glasdegib doses (e.g., for dose reductions) and exposures do not compromise the survival benefit of glasdegib 100 mg QD.

**Clinical Trial number:**

NCT01546038.

**Electronic supplementary material:**

The online version of this article (10.1007/s00280-020-04132-x) contains supplementary material, which is available to authorized users.

## Introduction

Glasdegib (PF-04449913) is a potent, selective, oral inhibitor of the Hedgehog signaling pathway. Based on the primary analysis of the BRIGHT AML 1003 trial, which demonstrated superior overall survival (OS) with glasdegib + low-dose cytarabine (LDAC) versus LDAC alone, glasdegib + LDAC was approved in the United States for the treatment of patients with newly diagnosed acute myeloid leukemia (AML) who are unable to receive intensive chemotherapy (ICT) as a result of comorbidities or older (≥ 75 years) age [1, 2]. Long-term (> 40 months) follow-up of BRIGHT AML 1003 showed a sustained, statistically significant improvement in OS among patients with AML receiving glasdegib + LDAC versus LDAC alone (hazard ratio [HR] 0.495, 95% confidence interval [CI] 0.325–0.752; *P* = 0.0004; median OS 8.3 vs. 4.3 months); the respective 2-year survival probability was 19.0 versus 2.8% [3]. The rate of complete remission was higher with glasdegib + LDAC versus LDAC alone (19.2 vs 2.6%) [4]. Glasdegib + LDAC treatment was associated with a reduced risk of cytopenias; with cycle 1 recovery of absolute neutrophil count (≥ 1000/µL, 45.1%), platelets (≥ 10 g/dL, 43.1%), and hemoglobin (≥ 100,000/µL, 33.3%) seen in a meaningful proportion of patients [5]. Additionally, more patients receiving glasdegib + LDAC (29.3%) were transfusion-independent vs. LDAC alone (5.6%) [6]. Long-term follow-up confirmed that the treatment combination was associated with an acceptable safety profile, with little additional toxicity (primarily related to nausea, and the inhibition of the Hedgehog signaling pathway [e.g., dysgeusia, muscle spasms]) [3] seen with glasdegib + LDAC versus LDAC alone. Currently, glasdegib, at a dose of 100 mg once daily (QD), is under further clinical evaluation in combination with a hypomethylating agent or ICT in patients with AML and myelodysplastic syndromes (MDS) [7, 8].

This population pharmacokinetic (PK)/pharmacodynamic (PD) analysis evaluated the time course of survival in patients with AML who were ineligible for ICT, comparing glasdegib + LDAC treatment relative to LDAC alone treatment (treatment–response), and explored the relationship between glasdegib exposure and OS (exposure–response). The effect of other covariates, including patient demographics, disease characteristics, and baseline laboratory values influencing OS probability were also investigated.

## Materials and methods

### Clinical studies

BRIGHT AML 1003 (ClinicalTrials.gov identifier: NCT01546038) was an open-label, randomized, multicenter, phase 1b/2 trial for which the methods have previously been published [1, 9]. Briefly, BRIGHT AML 1003 enrolled adult patients aged ≥ 55 years with newly diagnosed, previously untreated AML or high-risk MDS (World Health Organization 2008 classification), who were ineligible for ICT. The phase 1b portion evaluated glasdegib (100 or 200 mg QD) in combination with LDAC (Arm A) or decitabine (Arm B) [9]. In the phase 2 portion of the study, patients were randomized 2:1 to receive glasdegib 100 mg QD + LDAC or LDAC alone with OS as a primary efficacy endpoint [1]. Patients were followed for up to 4 years from the first dose. OS was defined as the date of randomization to the time of death for any reason. If death was not documented, censoring occurred at the date on which the subject was last known to be alive. Response to treatment was assessed based on the International Working Group response criteria guidelines for MDS and AML [10, 11]. The study was conducted in accordance with the Declaration of Helsinki. All patients provided written informed consent before study procedures began, and the protocol was approved by institutional review boards at each study site.

The population PK/PD analysis followed a prespecified analysis plan for data handling, model selection and evaluation, and testing of covariate effects.

### Study data

Using data from the phase 2 portion of the trial, the study population for the treatment–response analysis included all patients with AML who were enrolled in the glasdegib + LDAC or LDAC alone arm. The exposure–response analysis included a subset of patients with AML from the phase 2 glasdegib + LDAC arm who received at least one dose of glasdegib and had glasdegib PK information available.

An exploratory treatment–response analysis evaluating glasdegib in combination with a hypomethylating agent was also conducted, adding data from the phase 1b portion of the study in patients with AML (*n* = 5) and MDS (*n* = 2) who received glasdegib 100 or 200 mg QD with decitabine to the treatment–response analysis population (glasdegib + LDAC and LDAC alone in AML). The study data cut-off for all analyses was based on the primary completion date of 3 January 2017.

### Parametric time-to-event model for OS

All OS response endpoints were captured as events and non-events and, therefore, the models were developed using time-to-event (TTE) analyses. Parametric survival models were used to assess the relationship of OS with study treatment (treatment–response analysis) and with glasdegib exposure (exposure–response analysis), and to explore covariates. The TTE models for OS were developed from survival data using a cumulative hazard distribution function [12]. Constant or time-varying cumulative hazard distribution functions, including exponential, Weibull, and log-logistic distributions, were evaluated using the available data. The distribution that best fits the data was selected as the base model. All TTE analyses were performed using nonlinear mixed-effects modeling (NONMEM) software (version 7.3.0, ICON Development Solutions, Ellicott City, MD, USA).

### Covariate analysis

Based on clinical relevance, mechanistic plausibility, and visual inspection of the Kaplan–Meier Mean Covariate (KMMC) plots, potential covariates were selected and tested for significance. This was implemented using the stepwise covariate model (SCM) building procedure in Perl-speaks-NONMEM version 4.2.0 [13, 14]. In the KMMC methodology, the mean of each covariate was plotted for all individuals remaining in the study at every inflection point of the Kaplan–Meier OS curve. A strong relationship observed between a covariate and parameters of the TTE model suggested that the covariate influenced the OS curve. The mean value of a covariate that influenced OS would be expected to increase or decrease over time, whereas the mean value of a covariate that did not affect OS would be expected to remain constant over time [15].

Intrinsic and extrinsic variables (e.g., study treatment, demographic characteristics, disease characteristics, and baseline safety laboratory values) were evaluated, using the SCM approach, for inclusion in the base models of the treatment–response and exposure–response analyses. The SCM approach involved both forward addition and backward elimination with a significance level of *α* < 0.05 and *α* < 0.001, respectively.

Demographic covariates including baseline body weight, baseline age, sex, and race were tested. Disease characteristics tested included de novo or secondary disease, cytogenetic risk, prior treatment with hypomethylating agents, and baseline Eastern Cooperative Oncology Group performance status (ECOG PS). The following baseline laboratory tests and other factors were also evaluated: creatinine clearance, aspartate transaminase, white blood cells, percentage of bone marrow blasts, and percentage of peripheral blasts.

Categorical covariates were included in the base model using a linear model. Continuous covariates were evaluated using a linear, exponential, power, or maximal-effect model. The covariates were screened for pairwise correlation and the more clinically relevant covariate was selected to be included in the model. If a baseline covariate value was found to be missing and the covariate was measured at post-baseline visits, that value was then imputed using the value at the first available, or earliest, post-baseline visit. If a covariate value was entirely missing for the patient, the baseline value was imputed as the population median baseline value.

### Derivation of PK exposure metrics

To derive summary measures of glasdegib exposure, individual empirical Bayes estimates of PK parameters were generated from a previously described population PK model [16]. Because duration of treatment may significantly impact efficacy, glasdegib exposure metrics that were not time dependent or that were earlier in the treatment course were selected in the exposure–response analysis. The selected exposure metrics included: first dose maximum concentration (*C*_max_), end of cycle 1 *C*_max_, end of cycle 1 minimum concentration, cycle 1 cumulative area under the concentration–time curve (AUC), cycle 1 average concentration (*C*_avg_), average AUC over the dosing interval, and overall *C*_avg_. A cycle was defined as 28 days. *C*_avg_ was calculated by dividing AUC by time. Both the raw scale and natural log-transformed exposure metrics were tested through the SCM approach on the base model.

### Model evaluation

Model adequacy and predictive performance was evaluated based on change in objective function value, precision of parameter estimates, and graphical presentation of model-predicted Kaplan–Meier curves overlaid with observed Kaplan–Meier curves. The performance of the final model was evaluated by simulating survival data (*n* = 500) using parameter estimates from the final model and conducting a visual predictive check (VPC) [17, 18]. All post-processing graphical and statistical analyses were completed with R version 3.2.2 (R Foundation for Statistical Computing, Vienna, Austria).

## Results

### Summary of observed data

The treatment–response analysis in the AML subpopulation who were ineligible for ICT included 116 patients (glasdegib + LDAC, *n* = 78; LDAC alone, *n* = 38). Demographic and baseline data for patients with AML included in the treatment–response analysis are summarized by study treatment in Table 1. The median baseline age and weight of patients with AML were 76 years and 78.2 kg, respectively, and the majority of patients were male (*n* = 82, 71%) and white (*n* = 113, 97%). Median baseline safety laboratory values (e.g., creatinine clearance, aspartate transaminase, white blood cell count, percentage of bone marrow blasts, and percentage of peripheral blasts) were well matched between the two treatment groups. The median baseline creatinine clearance (calculated based on Cockcroft–Gault equation) was 62.7 mL/min, indicating that most patients had mild renal impairment (as defined by Kidney Disease Outcomes Quality Initiative classification) [19].

**Table 1:** Patient demographics and baseline characteristics by study treatment

Study	LDAC alone	Glasdegib + LDAC	All
*N*	38	78	116
Age, years	76 (58–83)	77 (64–92)	76 (58–92)
Weight, kg	78.6 (51.9–118.0)	78.2 (47.5–116.4)	78.2 (47.5–118.0)
Sex, male/female	23 (61)/15 (39)	59 (76)/19 (24)	82 (71)/34 (29)
Race			
White Black Asian	38 (100) 0 0	75 (96) 1 (1) 2 (3)	113 (97) 1 (1) 2 (2)
WBC, 10^9^ cells/L^a^	3.6 (1.2–1370.0)	2.8 (0.4–5850.0)	3.1 (0.4–5850.0)
Percentage bone marrow blasts^a^	48.3 (13–95)	41.5 (16–99)	44.0 (13–99)
Percentage peripheral blasts^a^	6.0 (0–85)	7.5 (0–91)	7.0 (0–91)
AST, U/L^a^	21.0 (7.0–111.0)	20.0 (8.0–73.0)	20.5 (7.0–111.0)
Creatinine clearance, mL/min^a^	68.2 (39.6–134.0)	60.0 (32.5–115.0)	62.7 (32.5–134.0)
Disease history			
De novo Secondary	18 (47) 20 (53)	38 (49) 40 (51)	56 (48) 60 (52)
Cytogenetic risk			
Good/intermediate Poor	21 (55) 17 (45)	49 (63) 29 (37)	70 (60) 46 (40)
Prior treatment			
No Yes	32 (84) 6 (16)	67 (86) 11 (14)	99 (85) 17 (15)
Baseline ECOG PS			
0 1 2 Missing	3 (8) 17 (45) 18 (47) 0	10 (13) 26 (33) 41 (53) 1 (1)	13 (11) 43 (37) 59 (51) 1 (1)

The data presented are median (min–max) for continuous variables and *n* (%) for categorical variables

*AST* aspartate transaminase, *ECOG PS* Eastern Cooperative Oncology Group performance status, *LDAC* low-dose cytarabine, *WBC* white blood cells

^a^For baseline laboratory results, all patients with baseline measurement are included

### Treatment–response analysis

The TTE treatment–response analysis base model was best described by a constant cumulative hazard distribution function. Evaluation of either the Weibull or the log-logistic models did not result in a statistically significant fit improvement compared with the exponential model. The full model was based on the forward inclusion of covariates at a significance level of *α* < 0.05. Treatment arm (e.g., glasdegib + LDAC or LDAC alone) and having poor cytogenetic risk factors were significant covariates influencing the base hazard based on the forward inclusion step of the SCM. However, treatment arm was the only covariate retained in the final model following the implementation of the backward elimination at the significance level of *α* < 0.001 (*P* = 0.000112), using the likelihood ratio test.

The probability of survival, *S*(*t*), at time *t* (in days) for the final model is described by the following equation:

$$S\left( t \right) = {\text{e}}^{{\left( { - 0.00253 \cdot \left( {1 + \left( {1.376 \cdot {\text{LDAC alone}}} \right)} \right) \cdot t} \right)}} ,$$

where the base hazard (relative standard error [RSE]) for glasdegib + LDAC was estimated at 0.00253 (13.82%). LDAC alone treatment resulted in an ~ 138% increase (multiply 1.376 by 1, or by 0 if not LDAC alone treatment) in base hazard (RSE, 37.74%), which equates to a shortening of median OS by approximately 5 months. The median HR was calculated to be 0.42 (95% CI 0.28–0.66) for glasdegib + LDAC treatment versus LDAC alone.

**Fig. 1:**
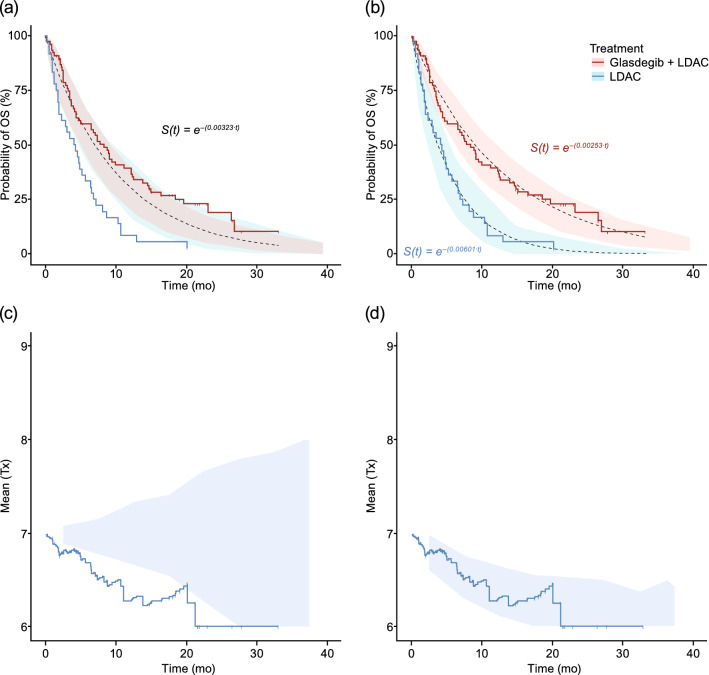
Treatment–response analysis of overall survival. The black dotted lines represent the survival functions, *S*(*t*) **a** from the base and **b** in final models with the 95% confidence interval of the predicted survival function in the shaded area by treatment arm. The solid lines are the observed OS data from the glasdegib + LDAC (pink) and LDAC alone (blue) treatment arms. KMMC plots for treatment arm for **c** the base and **d** final models. With the inclusion of treatment arm as a covariate in the final model, the KMMC plot was corrected. *KMMC* Kaplan–Meier mean covariate, *LDAC* low-dose cytarabine, *OS* overall survival, *S(t)* probability of survival, *Tx* treatment arm

Simulations of the base and final TTE treatment–response models were performed, and the VPC plots of survival by treatment arm are presented in Fig. 1. As depicted in the VPC plot generated for the base model (Fig. 1a), the survival function lies within the observed OS data of the glasdegib + LDAC and LDAC alone treatment arms. The VPC survival plot of the final model accounting for treatment differences (treatment arm as a covariate; Fig. 1b), adequately characterized the observed survival data, with the observed data overlapping the median predicted data and falling within the 95% prediction interval. The KMMC plots for the base and final models are presented in Fig. 1c, d. After inclusion of treatment arm as a significant covariate in the final model, the change in the mean for treatment in the direction of glasdegib + LDAC treatment was corrected.

### Exposure–response analysis

**Fig. 2:**
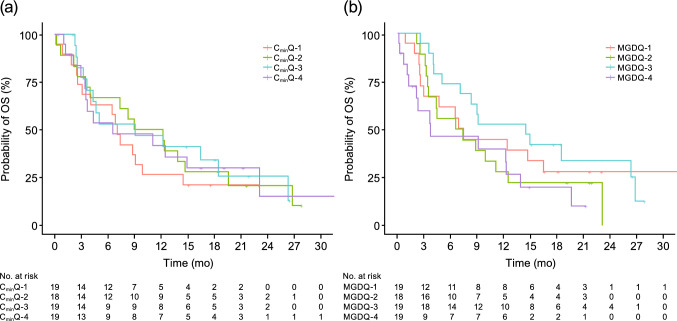
Kaplan–Meier plots for overall survival by **a** quartiles of cycle 1 glasdegib *C*_min_ and **b** quartiles of mean glasdegib daily dose (mg) over the treatment duration. *C*_*min*_*Q* minimum concentration quartile, *MGDQ* mean glasdegib dose quartile, *OS* overall survival

The TTE exposure–response analysis included a subset of the treatment–response analysis population who were randomized to glasdegib 100 mg QD and had available PK concentration data to derive glasdegib exposure metrics (*n* = 75). A summary of the glasdegib exposure metrics predicted from individual post-hoc estimates from the population PK model is presented in Table 2. During SCM, both the raw scale exposure metrics and log-transformed exposure metrics were evaluated. Kaplan–Meier plots of OS by quartiles of glasdegib exposure and dose metrics are presented in Fig. 2 and in the supplementary Fig., Online Resource 1. No apparent trends in quartiles of glasdegib exposure or dose were observed for OS.

**Table 2:** Summary of glasdegib exposure metrics (*N* = 75)

Exposure metric	Mean (SD)	Median (min–max)	25th percentile	75th percentile
First dose *C*_max_ (ng/mL)	592.0 (352.1)	602.2 (0.8–1437.8)	330.2	807.1
End of cycle 1 *C*_max_ (ng/mL)	1308.3 (729.3)	1139.9 (275.3–3612.5)	762.2	1603.2
Cycle 1 AUC (h ng/mL)	631,571.2 (356,987.3)	575,220.0 (54,368.0–1,797,600.0)	381,880	818,215
Cycle 1 *C*_avg_ (ng/mL)	1009.7 (556.2)	927.1 (161.9–3505.4)	596.9	1248.8
End of cycle 1 *C*_min_ (ng/mL)	750.7 (565.0)	577.6 (4.9–2762.0)	399.9	1055.0
Average AUC_tau_ (h ng/mL)	18,580.2 (11,135.7)	16,571.3 (2927.9–61,527.3)	11,682.3	22,979.8
Overall *C*_avg_ (ng/mL)	910.1 (535.2)	783.8 (218.5–3505.4)	553.6	1156.5

*AUC* area under the concentration–time curve, *AUC*_*tau*_ AUC for the dosing interval, *C*_*avg*_ average concentration, *C*_*max*_ maximum concentration, *C*_*min*_ minimum concentration, *SD* standard deviation

Similar to the treatment–response analysis, probability of OS was best described by an exponential model. During the forward selection step of SCM (*α* < 0.05), baseline ECOG PS and cytogenetic risk factors were covariates included in the full model. None of the exposure metrics reached the significance level in the forward inclusion step. Following backward elimination (*α* < 0.001), no covariates were determined to be statistically significant; therefore, the final model of the exposure–response analysis was the same as the base model. The estimated base hazard (RSE) was 0.00246 (14.19%) and the survival probability was described by:

$$S\left( t \right) = {\text{e}}^{{{-}0.00246 \cdot t}} .$$

**Fig. 3:**
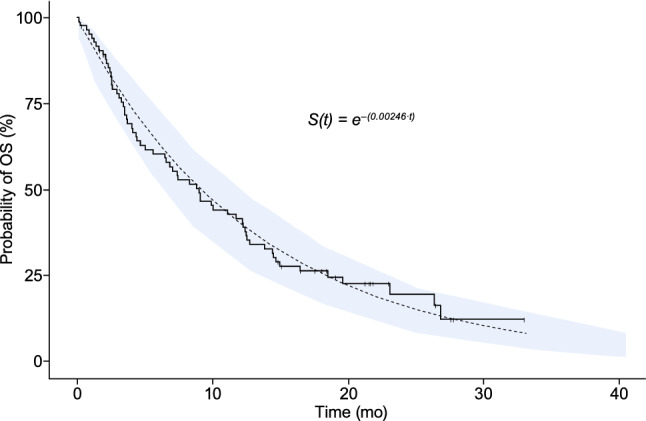
Exposure–response analysis of overall survival. The black dotted line represents the survival function, S(*t*) from the final model, with the 95% confidence interval of the predicted survival function in the shaded area. The solid line is the observed OS data from the exposure–response analysis data set. *OS* overall survival, *S(t)* probability of survival

A simulation of the final TTE exposure–response model was conducted, and the VPC plot of survival is presented in Fig. 3. The exponential model adequately characterized the observed survival data, with the observed data overlapping the predicted data and all within the 95% prediction interval.

### Exploratory analysis of glasdegib + decitabine treatment

An exploratory treatment–response analysis was also conducted to evaluate the relationship of combination therapy with glasdegib + decitabine and OS. This analysis included data from both patients with AML and MDS (*n* = 162) from Arms A (glasdegib + LDAC) and B (glasdegib + decitabine) of the phase 1b portion of the study, and from phase 2 patients (glasdegib + LDAC and LDAC alone) who were not indicated for ICT (as shown in the Table, Online Resource 2). The phase 1b Arm B portion included five patients with AML and two with MDS treated with glasdegib + decitabine.

The base TTE model was best described by an exponential model. In the full model following SCM, log transformation of baseline percentage of bone marrow blasts, prior use of hypomethylating agents, and treatment arm were included as covariates on the base hazard. Following backward elimination, only the effect of treatment arm was retained in the final model. The final model for this exploratory analysis was described by:

$$S\left( t \right) = {\text{e}}^{{{-}\left( {0.00540 \cdot \left( {1 - \left( {0.480 \cdot {\text{glasdegib}} + {\text{LDAC}}} \right)} \right) \cdot \left( {1 - \left( {0.618 \cdot {\text{glasdegib}} + {\text{decitabine}}} \right)} \right) \cdot t} \right)}} .$$

**Fig. 4:**
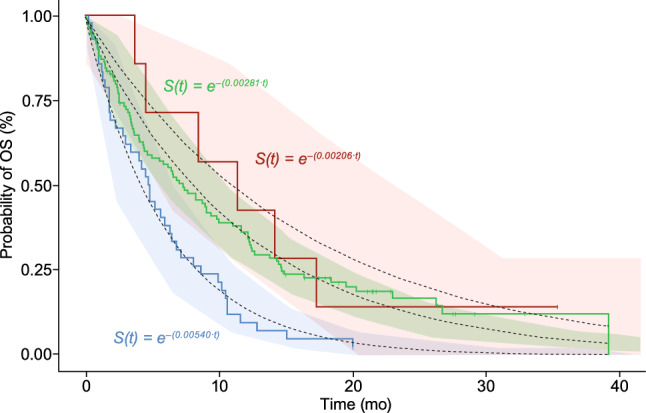
Exploratory treatment–response analysis including glasdegib + decitabine treatment. The black dotted lines represent the survival functions, *S*(*t*) from the exploratory treatment–response final model with the 95% confidence interval of the predicted survival function in the shaded area by treatment arm. The solid lines are the observed OS data from the glasdegib + decitabine (pink), glasdegib + LDAC (green), and LDAC alone (blue) treatment arms. *LDAC* low-dose cytarabine, *OS* overall survival, *S(t)* probability of survival

The base hazard was estimated at 0.00540 (14.1% RSE) for the reference treatment, LDAC alone. For the glasdegib + decitabine treatment, the base hazard was approximately 61.8% lower than the base hazard for LDAC alone, with an estimated median OS of 11.1 months for glasdegib + decitabine. Due to the small sample size of the patients treated with glasdegib + decitabine, the CI of the change in base hazard relative to the LDAC alone treatment was wide (– 95.0 to – 28.6%) and the 95% prediction interval of the simulated survival data for this treatment group was also wide (Fig. 4). The prediction intervals for glasdegib + decitabine fully encompassed the prediction interval for glasdegib + LDAC and slightly overlaps with the LDAC alone treatment at the later timepoints (> 20 months) where the sample size was small.

## Discussion

This study characterized the time course of survival with glasdegib + LDAC relative to LDAC alone (treatment–response) and explored whether the differences in glasdegib exposure at the dose of 100 mg QD significantly affected OS (exposure–response) based on data from the BRIGHT AML 1003 trial in patients with newly diagnosed AML who were ineligible for ICT. For both the treatment–response and exposure–response study populations, the survival function was best characterized by an exponential TTE distribution model.

The treatment–response analysis indicated that treatment arm (glasdegib + LDAC or LDAC alone) had a statistically significant impact on OS. The addition of glasdegib to LDAC resulted in a 58% reduction in the risk of death, translating to a median OS prolonged by approximately 5 months (HR 0.42, 95% CI 0.28–0.66). These results are similar to those reported from the primary analysis (the same data cut-off used in this analysis) of BRIGHT AML 1003 using a Cox proportional hazards model in patients with AML (HR 0.46, 95% CI 0.30–0.71; median OS 8.3 vs. 4.3 months) [2]. Together these results support the survival benefit of glasdegib + LDAC (vs. LDAC alone) in the treatment of patients with newly diagnosed AML ineligible for ICT.

In the exploratory treatment–response analysis in patients with AML and MDS, glasdegib + decitabine treatment had an estimated 61.8% base hazard reduction from the standard-of-care therapy, LDAC alone. Although the sample size in the exploratory analysis with glasdegib + decitabine was small, the estimated OS of 11.1 months compares favorably to the observed clinical data (median OS, 11.5 months) and the historically reported median OS of 7.7 months for decitabine monotherapy [9, 20]. A randomized, double-blind, multicenter, placebo-controlled phase 3 trial (BRIGHT AML 1019; ClinicalTrials.gov identifier: NCT03416179) of glasdegib in combination with azacitidine in patients with newly diagnosed AML is ongoing. The choice of azacitidine as the combination agent was based on pre-clinical evidence of synergistic effect between a Smoothened inhibitor and azacitidine, and experience from another phase 1b clinical trial involving dosing of glasdegib plus azacitidine in patients with AML and MDS (BRIGHT 1012; NCT02367456) [21, 22].This trial also includes a second randomized, double-blind, placebo-controlled cohort investigating glasdegib in combination with ICT in patients with newly diagnosed AML [8].

In the phase 2 portion of BRIGHT AML 1003, all patients in the glasdegib + LDAC arm were randomized to receive glasdegib 100 mg QD and were permitted to reduce the glasdegib dose for the management of adverse events (AEs). The exposure–response analysis demonstrated that variability in glasdegib exposures at the 100 mg QD dose did not impact the risk of death or the OS curves in patients with AML. Therefore, the survival benefit of glasdegib + LDAC was determined not to be glasdegib exposure–dependent; however, these results are limited by the availability of only one glasdegib dose level in BRIGHT AML 1003 (100 mg QD). Long-term follow-up of BRIGHT AML 1003 in patients with AML confirmed that glasdegib + LDAC was well tolerated [6]. However, some patients may require dose modifications to manage the occurrence of AEs; the most common AEs associated with glasdegib + LDAC treatment in the first 90 days and after 90 days were anemia and diarrhea, respectively. In the glasdegib + LDAC arm, 14/75 (18.7%) patients had glasdegib dose reduced at any time on study (data unpublished). Of these, 13/75 (17.3%) patients had glasdegib dose reductions due to treatment-related AEs (data unpublished). The proportion of patients needing dose reduction at the 100 mg QD dose is considered low. The exposure–response analysis suggests that the management of AEs with dose reduction of glasdegib may allow patients with AML to remain on treatment without impacting the survival benefit of glasdegib + LDAC.

In both the treatment–response and exposure–response analyses, demographic characteristics (e.g., age, sex, baseline weight, race) and baseline safety laboratory values were evaluated as potential sources of variability affecting OS, but none were significant covariates on the base hazard. Additionally, baseline disease characteristics (ECOG PS, de novo or secondary disease, cytogenetic risk, and prior use of hypomethylating agents) were also explored as potential covariates, but none of these characteristics impacted the probability of an event that would modify the OS curves. These results demonstrate that the survival benefit associated with glasdegib treatment is independent of patient demographics, baseline safety laboratory values, and baseline disease characteristics, and support the broad use of glasdegib 100 mg QD in combination with chemotherapy in patients who are ineligible to receive ICT.

In conclusion, the addition of glasdegib to LDAC chemotherapy resulted in significant OS benefit in patients with AML who were ineligible to receive ICT. The survival function was best characterized by an exponential TTE distribution model. The addition of glasdegib to LDAC chemotherapy resulted in a 58% reduction in the risk of death. Variability in glasdegib exposures, demographics, baseline safety laboratory values, and disease characteristics did not impact the probability of an event modifying the OS curves. Together these results support the broad use of glasdegib 100 mg QD with chemotherapy in the treatment of this AML subpopulation.

## Electronic supplementary material

Below is the link to the electronic supplementary material.

Supplementary file1 (DOCX 198 kb)

## Data Availability

Upon request, and subject to certain criteria, conditions and exceptions (see https://www.pfizer.com/science/clinical-trials/trial-data-and-results for more information), Pfizer will provide access to individual de-identified participant data from Pfizer-sponsored global interventional clinical studies conducted for medicines, vaccines, and medical devices: (1) for indications that have been approved in the US and/or EU; or (2) in programs that have been terminated (i.e., development for all indications has been discontinued). Pfizer will also consider requests for the protocol, data dictionary, and statistical analysis plan. Data may be requested from Pfizer trials 24 months after study completion. The de-identified participant data will be made available to researchers whose proposals meet the research criteria and other conditions, and for which an exception does not apply, via a secure portal. To gain access, data requestors must enter into a data access agreement with Pfizer.
