# Supplementary material for: An evaluation of overall survival in patients with newly diagnosed acute myeloid leukemia and the relationship with glasdegib treatment and exposure
Source: Cancer Chemother Pharmacol. 2020 Sep 3;86(4):451–9. doi: 10.1007/s00280-020-04132-x (PMC7515941; doi:10.1007/s00280-020-04132-x)
Supplement: Supplementary file 1 — Supplementary file1 (DOCX 198 kb) [file 280_2020_4132_MOESM1_ESM.docx]

**Supplementary Materials**

**Online resource 1** Kaplan–Meier plots of overall survival by (**a**) quartiles of glasdegib cycle 1 AUC and (**b**) quartiles of glasdegib cycle 1 C_max_. *AUC* area under the concentration–time curve, *AUCQ* AUC quartile, *C_max_* maximum concentration, *C_max_Q* C_max_ quartile, *OS* overall survival


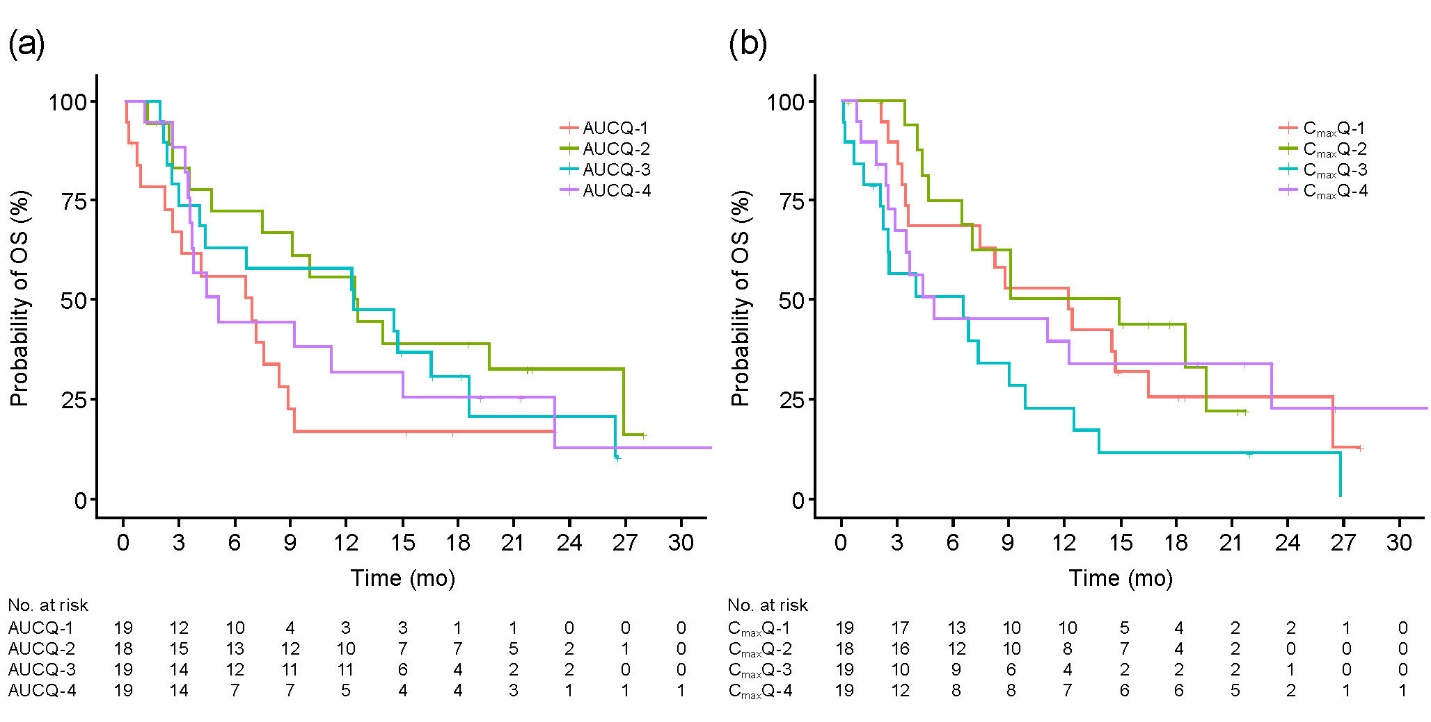


**Online resource 2** Number of patients in each treatment arm, by disease setting and study phase

| Treatment arm | Study phase | Disease setting | Patients, *n* |
| --- | --- | --- | --- |
| Glasdegib + LDAC | 1b | AML | 20 |
| Glasdegib + LDAC | 1b | MDS | 3 |
| Glasdegib + decitabine | 1b | AML | 5 |
| Glasdegib + decitabine | 1b | MDS | 2 |
| Glasdegib + LDAC | 2 | AML | 78 |
| Glasdegib + LDAC | 2 | MDS | 10 |
| LDAC alone | 2 | AML | 38 |
| LDAC alone | 2 | MDS | 6 |

*AML* acute myeloid leukemia, *LDAC* low-dose cytarabine, *MDS* myelodysplatic syndrome
